# Removal of emerging contaminants in a full-scale bioreactor coupled with membrane filtration for reclaimed water production in São Paulo State, Brazil

**DOI:** 10.1007/s11356-026-37765-1

**Published:** 2026-04-29

**Authors:** Rafaela Gonçalves Machado, Josilei da Silva Ferreira, Guilherme Martins Grosseli, Roberta Cerasi Urban, Pedro Sergio Fadini

**Affiliations:** Environmental Biogeochemistry Laboratory and Center for Environmental Diagnostics and Interventions, Department of Chemistry, Federal University of São Carlos, São Carlos, Brazil

**Keywords:** Emerging contaminants, Reclaimed water, Membrane bioreactor, Wastewater treatment, Pharmaceutical residues, Water quality, Endocrine disruptors

## Abstract

Water scarcity and water quality degradation have increased the interest in reclaimed water production from wastewater treatment plants. Among quality concerns, emerging contaminants (ECs) have gained attention due to their persistence and potential environmental and health risks. This study investigated the occurrence, spatial distribution, and removal of ECs in a full-scale Water Resource Recovery Facility (WRRF) operating an anaerobic–anoxic–aerobic process followed by membrane bioreactor (MBR) filtration in São Paulo State, Brazil. Wastewater samples were collected at different treatment stages, and surface waters from regional rivers were analyzed for comparison. Target compounds were determined by UPLC–MS/MS and GC–MS. All analytes were detected in raw sewage, with caffeine presenting the highest concentrations. High removal efficiencies were observed for paracetamol, caffeine, ibuprofen, naproxen, and atenolol (> 97%), while diclofenac and propranolol showed moderate removal (60–70%). Estrogenic compounds exhibited variable removal, and carbamazepine and its metabolites showed persistent behavior, with negative removal suggesting back-formation processes. The results confirm carbamazepine as a reliable chemical marker of treatment efficiency and indicate that, although reclaimed water showed high overall quality, complementary treatment may be required to meet stricter standards for emerging contaminants.

**Supplementary Information:**

The online version contains supplementary material available at 10.1007/s11356-026-37765-1.

## Introduction

The rapid and unplanned growth of the global population, particularly in large urban centers, combined with technological development and intense industrialization, has significantly impacted water resources through both excessive consumption and environmental degradation caused by the improper disposal of solid and liquid wastes. These factors, along with recurrent and severe droughts, have led to water scarcity in many regions worldwide, posing a major contemporary challenge: balancing increasing demand with limited water availability. In this context, the production of reclaimed water from wastewater treatment has become an increasingly encouraged practice (Kahn et al. 2025; Yoon et al. 2024).

Properly planned and managed water reuse systems can generate significant benefits for both the environment and society. They can reduce the discharge of insufficiently treated wastewater into water bodies, preserve overexploited groundwater sources, and can contribute, especially in developing countries, to increased food production, improved public health, and enhanced quality of life and social conditions in communities involved in reuse programs (Hajjar et al. 2025; Yan et al. 2014). However, concerns regarding water resources extend beyond availability to include water quality. In this regard, a class of pollutants known as emerging contaminants (ECs) has gained growing attention from the scientific community due to their frequent detection in various environmental compartments and in effluents from sewage treatment plants (STPs) (Kumar et al. 2022; Ferronato and Torretta 2019).

Emerging contaminants comprise thousands of chemical substances of both natural and synthetic origin that have been detected in environmental matrices; however, their effects on the environment and human health remain poorly understood (Morin-Crini et al. 2022; Wu et al. 2019). Currently, most of these compounds are not included in routine monitoring programs or environmental legislation, mainly due to the lack of data on ecotoxicity, bioaccumulation potential, and long-term effects on human health (Montagner et al. 2017).

In aquatic environments, many ECs are considered pseudo-persistent due to their continuous introduction into water bodies. In addition, compounds with low biodegradability can cause adverse impacts on aquatic biota, including genotoxicity, endocrine disruption, and acute or chronic aquatic toxicity. Moreover, degradation processes may generate transformation products with even more deleterious effects compared to the parent compounds (Agarwal et al. 2025).

Given the harmful effects of these contaminants on aquatic environments, and considering that sewage treatment plants (STPs) are recognized as the main point sources of ECs in receiving waters, there is a pressing need to produce high-quality effluents. Conventional treatment systems, however, were not designed to remove micropollutants typically present at trace concentrations (μg L⁻^1^ to ng L⁻^1^) (Kahn et al. 2025; Agarwal et al. 2025; Marasco Júnior et al. 2019). Understanding the occurrence, fate, and behavior of ECs in STPs can help identify compounds that serve as chemical markers of treatment efficiency. Chemical markers are typically compounds that exhibit high detection frequency, environmental persistence, low seasonal variability, and source specificity, allowing them to be used as indicators of treatment performance and potential contamination pathways in receiving waters. Identifying such markers can help ensure that discharged water does not cause significant ecological impacts or, alternatively, that it can be safely reused to mitigate water scarcity.

Therefore, this study investigates the occurrence, spatiotemporal distribution, and removal of emerging contaminants in a full-scale reclaimed water production plant located in São Paulo State, Brazil, operating with a membrane bioreactor (MBR) coupled to an anaerobic–anoxic–aerobic (A2O) process. The novelty of this study lies in the evaluation of a full-scale A2O + MBR system under Brazilian conditions, including not only parent compounds but also metabolites and estrogenic compounds, as well as in the assessment of contaminant behavior across different treatment stages. In addition to investigating the occurrence and removal patterns of these compounds throughout the treatment process, this study also seeks to identify potential chemical markers useful for evaluating wastewater treatment performance and to compare the quality of the reclaimed water produced by the WRRF with that of surface waters used as drinking water sources in the same region.

In this context, the present study was guided by the hypothesis that the removal of emerging contaminants in the investigated WRRF is influenced by both the treatment stage and the physicochemical and biodegradation properties of each compound. Compounds with high biodegradation potential, such as caffeine and paracetamol, were expected to show high removal efficiencies during biological treatment, whereas more persistent compounds, such as carbamazepine and its metabolites, were expected to exhibit limited removal and remain detectable throughout the treatment process. In addition, differences in removal behavior among the investigated contaminants were hypothesized to be associated with parameters such as pKa, log Kow, and Kbio, which influence sorption and biodegradation during wastewater treatment.

## Experimental

### Sampling area

After preliminary treatment, the sewage is directed to biological reactors, where different operational conditions promote the removal of carbonaceous organic matter and nutrients. The mixed liquor resulting from the biological process is transferred to MBR tanks, where ultrafiltration takes place. The generated permeate, referred to as reclaimed water, is stored in a reservoir until it is pumped into tanker trucks, with the surplus discharged into a river. The MBR system employs hollow-fiber membranes with a nominal pore size of 0.04 µm and a total filtration area of approximately 72,000 m^2^.

**Fig. 1:**
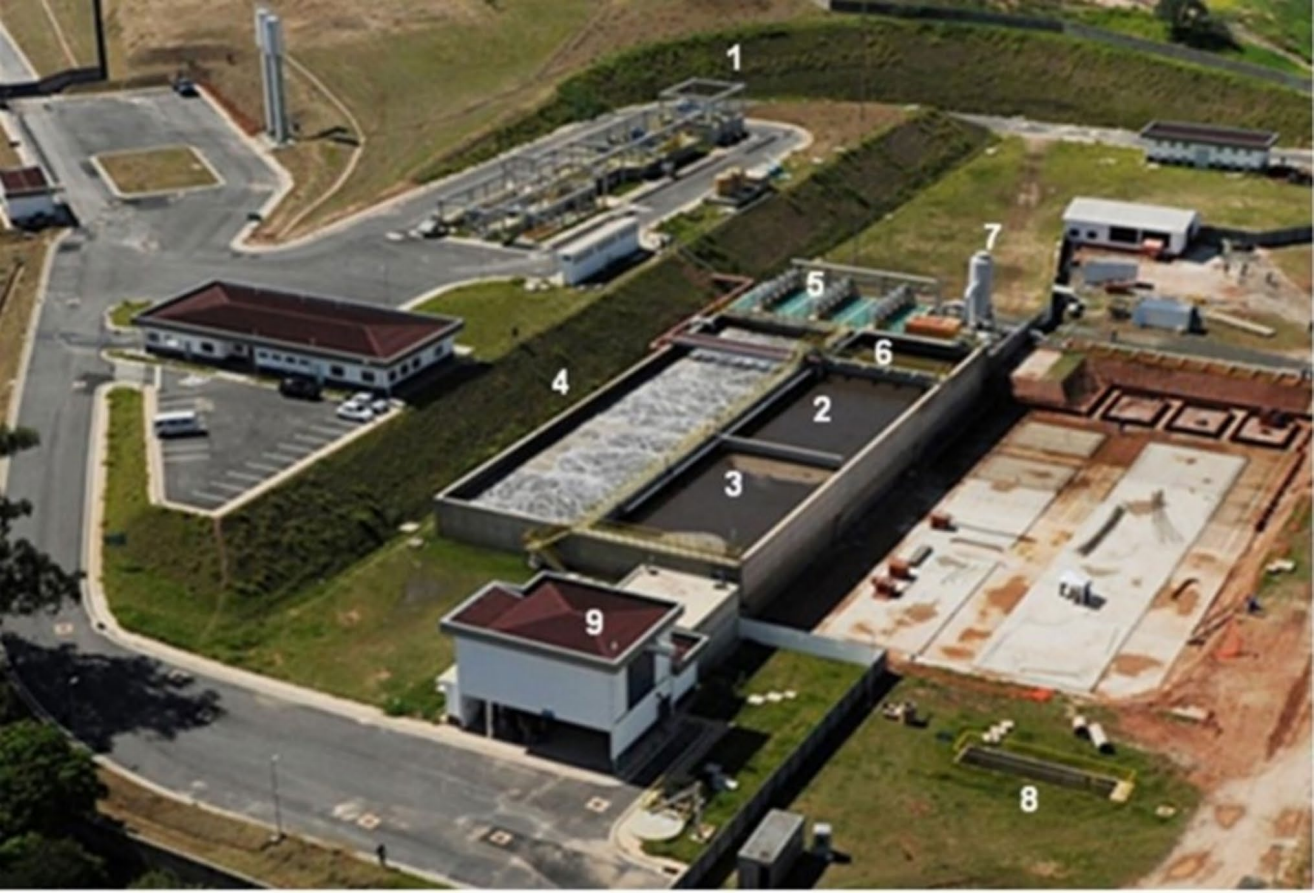
Aerial view of the Water Resource Recovery Facility (WRRF). Units: (1) preliminary treatment, (2) anaerobic tank, (3) anoxic tank, (4) aeration tank, (5) membrane tank, (6) deoxygenation tank, (7) treated effluent tank, (8) Parshall flume, and (9) sludge dewatering building

Sampling was conducted at six different points within the WRRF, as illustrated in Fig. 1: (1) 24 h composite sample of raw sewage (WRRF inlet), (2) grab sample from the anaerobic tank, (3) grab sample from the anoxic tank, (4) grab sample from the aeration tank, (5) grab sample from the MBR tank, and (6) 24 h composite sample of treated effluent/reclaimed water (WRRF outlet). In addition to the aqueous samples, sludge samples were also collected from the aeration tank for sorption assays. All collected samples were kept under refrigeration during transport to the laboratory.

Sampling campaigns were conducted during multiple monitoring events (*n* = 14 for most compounds and *n* = 11 for estrogens), allowing the evaluation of temporal variability in the occurrence and removal of emerging contaminants in the WRRF. Precipitation data for the study area were evaluated for the sampling days, and no significant variations in contaminant concentrations associated with rainfall events were observed during the monitoring campaigns. The treatment plant operates under relatively stable hydraulic and operational conditions, serving approximately 175,000 inhabitants with an average flow rate of approximately 360 L s⁻^1^ and a hydraulic retention time of 6 h; therefore, the monitoring campaigns allowed the observation of consistent patterns in contaminant occurrence and removal under the operational conditions of the WRRF.

It should be noted that influent and treated effluent samples were collected as 24 h composite samples to obtain representative average concentrations of the wastewater entering and leaving the treatment plant. Composite sampling is commonly used in wastewater treatment studies because it integrates daily variations in sewage load associated with the activities of the served population. In contrast, grab samples were collected from the anaerobic, anoxic, aeration, and MBR tanks to characterize contaminant behavior along the different treatment units. In this study, removal values reported for each tank were calculated relative to the concentrations observed in the raw sewage, allowing the evaluation of reduction trends along the treatment system. Therefore, the removal profiles should be interpreted as indicative patterns of contaminant behavior across the treatment units rather than strictly time-synchronized mass balances between process stages.

Additionally, surface water samples were collected from the Atibaia, Capivari, Jundiaí, and Ribeirão Piraí rivers, located in the same region of São Paulo State, for quality comparison with the reclaimed water produced by the WRRF.

### Chemical reagents

The target compounds, caffeine (CAF), paracetamol (PAR), naproxen (NPX), diclofenac (DCL), ibuprofen (IBU) and its metabolites (1-hydroxyibuprofen, IBU-1OH, and 2-hydroxyibuprofen, IBU-2OH), atenolol (ATL), propranolol (PRP), carbamazepine (CBZ) and its metabolites (2-hydroxycarbamazepine, CBZ-2OH, and 10,11-dihydro-10,11-dihydroxycarbamazepine, CBZ-DIOH), estrone (E1), 17β-estradiol (E2), and 17α-ethinylestradiol (EE2), were purchased from Dr. Ehrenstorfer (Augsburg, Germany) with purity > 96%.

Isotopically labeled internal standards (caffeine-d_3_, paracetamol-d_3_, naproxen-d_3_, diclofenac-d_4_, ibuprofen-d_3_, atenolol-d_7_, propranolol-d_7_, carbamazepine-d_10_, estrone-d_4_, 17β-estradiol-d_5_, and 17α-ethinylestradiol-d_4_) were obtained from CDN Isotopes (Quebec, Canada). The selected compounds represent pharmaceuticals with high consumption rates and frequent detection in aquatic environments worldwide.

HPLC-grade solvents, including methanol and acetone, were supplied by Sigma-Aldrich (Switzerland). Ultrapure water was obtained using a Milli-Q purification system (Millipore, Merck Millipore, São Paulo, Brazil). HPLC-grade ammonium hydroxide was also purchased from Sigma-Aldrich (Switzerland). Nitrogen gas (99.9% purity) used for evaporation was supplied by White Martins (São Paulo, Brazil).

### Sample preparation

Samples collected in amber polyethylene terephthalate (PET) bottles were vacuum-filtered through 1.2 µm and 0.7 µm glass fiber filters, followed by 0.45 µm nylon membrane filters (Sartorius, Göttingen, Germany). Solid-phase extraction (SPE) was performed using Oasis HLB cartridges (200 mg, 6 cm^3^) supplied by Waters Corporation (Milford, USA), following the method developed by Sousa et al. (2014). Cartridges were preconditioned with 2 × 5 mL of methanol and 2 × 5 mL of ultrapure water using a vacuum manifold system.

A 100 mL aliquot of each sewage sample was spiked with 100 ng L⁻^1^ of each internal standard and passed through the cartridges at a flow rate of 5 mL·min⁻^1^. The cartridges were then washed with ultrapure water (3 × 15 mL) and dried under vacuum for 40 min. Elution was carried out with 2 × 3 mL of methanol followed by 3 mL of methanol:acetone (1:1, *v*/*v*). Extracts were evaporated under a gentle nitrogen stream using a Dry-Block SL-22/50 system (Solab Equipamentos para Laboratório Ltda, Piracicaba, Brazil) and reconstituted with 1 mL of methanol:water (20:80, *v*/*v*). All samples were extracted and analyzed in triplicate.

For surface water samples, the same extraction method (de Sousa et al. 2014) was applied, except that a smaller washing volume (2 × 4 mL ultrapure water) and a larger sample volume (500 mL) were used. After reconstitution, aliquots were separated for estrogen analysis by GC–MS and for other analytes by LC–MS/MS, with all samples analyzed in triplicate.

For sludge samples collected from the aeration tank, the sludge was first dried and sieved. Then, 100 mg of the processed sludge was weighed and spiked with internal standards to correct for analytical variability during chromatographic analysis. The mixture was allowed to stand for 24 h, and all extractions were performed in triplicate. Extraction was carried out with 2 mL of a methanol:water solution (50:50, *v*/*v*) adjusted to pH 2.5 with 0.5% formic acid and containing 1% EDTA. The sample was vortex-mixed and then subjected to ultrasonic extraction for 15 min at 50 °C. After extraction, the sample was centrifuged for 10 min, and the supernatant was collected in a glass test tube. This procedure was repeated three times, resulting in a total extract volume of 6 mL. The combined extract was evaporated to dryness under a gentle nitrogen stream in a Dry-Block system at 40 °C. The analytes were then reconstituted in 1 mL of methanol:water containing 0.05% formic acid (25:75, *v*/*v*), following Gago-Ferrero et al. (2015) (Gago-Ferrero et al. 2015). Finally, the extract was filtered through a 0.2 µm syringe filter, and the final volume was divided equally for pharmaceutical analysis by LC–MS/MS and hormone analysis by GC–MS.

### Analysis of emerging contaminants

Emerging contaminants were analyzed using ultra-performance liquid chromatography coupled to a triple-quadrupole mass spectrometer (Waters TQD) with electrospray ionization (ESI). Chromatographic separation was performed on an ACQUITY UPLC BEH C18 column (2.1 × 50 mm, 1.7 µm particle size) using a method optimized in this study. The mobile phase consisted of (A) ultrapure water containing 0.05% (*v*/*v*) ammonium hydroxide and (B) methanol, with the following gradient: 0–2.3 min (90% A, 10% B); 2.3–3.5 min (55% A, 45% B); 3.5–6.0 min (5% A, 95% B); 6.0–6.5 min (5% A, 95% B); 6.5–6.6 min (90% A, 10% B); and 6.6–8.5 min (90% A, 10% B). The injection volume was 5 µL, the flow rate was 0.35 mL·min⁻^1^, and the column temperature was maintained at 40 °C.

The mass spectrometer operated in both positive and negative ionization modes, with capillary voltages of 3000 V and 2500 V, respectively. Nitrogen was used as both the nebulizing gas (20 L h⁻^1^) and the desolvation gas (750 L h⁻^1^). The desolvation and source block temperatures were set at 500 °C and 150 °C, respectively. The instrument operated in Selected Reaction Monitoring (SRM) mode, monitoring the most abundant precursor-to-product ion transitions for each analyte.

Estrogens (estrone, 17β-estradiol, and 17α-ethinylestradiol) were determined by gas chromatography–mass spectrometry (GC–MS). Prior to analysis, samples were derivatized by evaporating 100 µL of extract and reconstituting it with 75 µL of N,O-bis(trimethylsilyl)trifluoroacetamide and 25 µL of pyridine. The mixture was incubated for 1 h at 80 °C and then injected into the chromatograph. The analysis was performed on a Shimadzu QP2010 GC–MS system equipped with an SLB-5 ms capillary column (30 m × 0.25 mm × 0.25 µm, 5% diphenyl/95% dimethylpolysiloxane, Sigma-Aldrich). Helium was used as the carrier gas at a flow rate of 2 mL min⁻^1^, with injector and ion source temperatures of 300 °C and 250 °C, respectively. The oven temperature program started at 100 °C (1 min), increased at 4 °C min⁻^1^ to 300 °C, and was held for 55 min. The mass spectrometer operated in electron impact (EI) mode at 70 eV with selected ion monitoring (SIM).

Quality assurance (QA) and quality control (QC) procedures included recovery assays, replicate analyses, calibration curve evaluation, and determination of method detection and quantification limits. Absolute and relative recovery experiments were performed in triplicate using treated effluent samples and sludge samples collected from the aeration tank. Calibration curves for LC–MS/MS and GC–MS analyses were prepared by internal standardization over the concentration range of 0.05–1200 µg L⁻^1^ (17 points), with internal standards maintained at a constant concentration of 50 µg L⁻^1^ throughout the analyses. Linearity was evaluated using the coefficient of determination (R^2^), and values above 0.99 were considered acceptable. Limits of detection (LOD) and quantification (LOQ) were estimated from the calibration curve parameters. Quantification was performed using internal standards, and final analyte concentrations were corrected according to the recovery values obtained during method validation. For LC–MS/MS identification, quantification was based on two selected reaction monitoring (SRM) transitions per compound, according to European Commission Decision 2002/657/EC. The most intense transition was used for quantification and the second for confirmation, and identification was based on retention time agreement and ion-ratio consistency relative to calibration standards. Detailed information on the validation procedure, as well as the results obtained for the evaluated analytical parameters, including recovery, linearity, LOD, and LOQ, is provided in the Supplementary Material (Tables S1–S3).

### Data treatment and removal calculation

Removal percentages were calculated according to Eq. (1), using the concentration measured in the raw sewage as reference:

1 $$\mathrm{Removal}\;\left(\%\right)=\left(\frac{C_{raw\;sewagw}-C_{tank}}{C_{raw\;sewage}}\right)\times100$$

where *C*_*raw sewage*_ represents the concentration measured in the influent wastewater and *C*_*tank *_corresponds to the concentration measured in each treatment tank or in the treated effluent.

Therefore, the removal values reported for the different treatment units represent reductions relative to the raw sewage concentration rather than sequential stage-by-stage removal efficiencies. Concentrations reported as below the detection limit (< LOD) were treated as zero for the purpose of removal calculations.

## Results and discussion

### The occurrence of emerging contaminants in the raw sewage

**Fig. 2:**
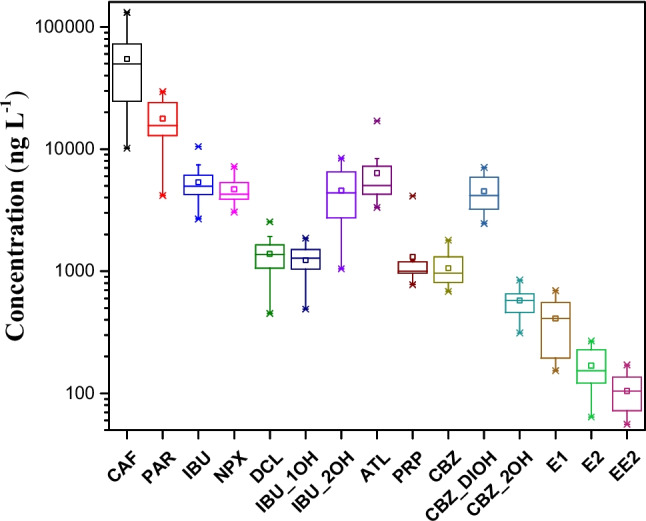
Concentrations of emerging contaminants in raw sewage. CAF: caffeine; PAR: paracetamol; IBU: ibuprofen; NPX: naproxen; DCL: diclofenac; IBU_2OH: 2-hydroxyibuprofen; IBU_1OH: 1-hydroxyibuprofen; ATL: atenolol; PRP: propranolol; CBZ: carbamazepine; CBZ_DIOH: 10,11-dihydro-10,11-dihydroxycarbamazepine; CBZ_2OH: 2-hydroxycarbamazepine; E1: estrone; E2: 17β-estradiol; EE2: 17α-ethinylestradiol**.** Box plots represent the distribution of concentrations measured in raw sewage during the monitoring campaigns (*n* = 14 for pharmaceuticals and metabolites; *n* = 11 for estrogens). The central line indicates the median, the box represents the interquartile range (IQR), and the whiskers correspond to the minimum and maximum values. Symbols indicate outliers. The *y*-axis is presented on a logarithmic scale

The contaminants investigated were detected in all raw sewage samples. The concentrations of each compound are shown in Fig. 2 as boxplots.

Caffeine was the compound quantified at the highest concentrations in raw sewage, ranging from 14,379 to 131,358 ng L⁻^1^, with a median (Md) of 56,709 ng L⁻^1^. The elevated concentrations of caffeine may be attributed to the widespread consumption of beverages, foods, and pharmaceuticals containing this compound (Marasco Júnior et al. 2019).

Among the anti-inflammatory and analgesic drugs, paracetamol was found at the highest concentrations (4166–29,491 ng L⁻^1^; Md = 18,326 ng L⁻^1^), followed by ibuprofen (3274–11,314 ng L⁻^1^; Md = 5774 ng L⁻^1^) and naproxen (3053–6899 ng L⁻^1^; Md = 4537 ng L⁻^1^). Diclofenac was detected at lower concentrations (451–2537 ng L⁻^1^; Md = 1405 ng L⁻^1^). In addition to ibuprofen, its metabolites were also quantified, with higher concentrations for 2-hydroxyibuprofen (2068–11,319 ng L⁻^1^; Md = 5839 ng L⁻^1^), which is consistent with its higher excretion rate (28%) compared to 1-hydroxyibuprofen (490–1952 ng L⁻^1^; Md = 1405 ng L⁻^1^), whose excretion rate is approximately 6% (Magiera and Gülmez 2014).

The β-blockers atenolol and propranolol, commonly prescribed for hypertension, were found at concentrations ranging from 3287 to 16,991 ng L⁻^1^ (Md = 6687 ng L⁻^1^) and 591 to 4135 ng L⁻^1^ (Md = 1196 ng L⁻^1^), respectively. The higher concentrations of atenolol may be associated with its limited metabolism, as approximately 90% of the administered dose is excreted unchanged in urine and feces, whereas only about 1% of propranolol is eliminated unchanged (Khan and Mahmood 2019).

Carbamazepine, an anticonvulsant commonly used for long-term treatment, showed concentrations between 587 and 1860 ng L⁻^1^ (Md = 1065 ng L⁻^1^). However, this compound is also excreted as glucuronide conjugates and metabolites, with 10,11-dihydro-10,11-dihydroxycarbamazepine being the major metabolite (2456–7079 ng L⁻^1^; Md = 4517 ng L⁻^1^), followed by 2-hydroxycarbamazepine (312–890 ng L⁻^1^; Md = 579 ng L⁻^1^) (Bahlmann et al. 2014).

Regarding estrogens, estrone was detected in concentrations ranging from 153 to 694 ng L⁻^1^ (Md = 410 ng L⁻^1^), while 17β-estradiol ranged from 64 to 268 ng L⁻^1^ (Md = 168 ng L⁻^1^). The synthetic estrogen 17α-ethinylestradiol was also quantified (56–171 ng L⁻^1^; Md = 105 ng L⁻^1^). The higher concentrations of estrone in raw sewage are attributed to its greater daily excretion by both men and women (Liu et al. 2015). These elevated levels may also be linked to the conversion of 17β-estradiol and 17α-ethinylestradiol into estrone during the transport of wastewater to the treatment plant (Czajka and Londry 2006).

### The behavior of the contaminants investigated during sewage treatment

The removal mechanisms of emerging contaminants in sewage treatment plants are influenced by several factors, including the physicochemical properties of the micropollutants, the configuration of the treatment systems, and the operational parameters adopted (Kofi Yeboah Adjei et al. 2025).

Sorption and biodegradation are the main mechanisms responsible for the removal of organic micropollutants in treatment plants equipped with an MBR system. Volatilization is generally negligible for pharmaceutical compounds, and photodegradation is considered insignificant due to the high concentration of sludge, which limits sunlight penetration (Gurung et al. 2019).

In the sorption process, mass transfer occurs when pollutants in the aqueous phase associate with a solid matrix (biomass and/or suspended solids). The extent of this phenomenon can be estimated using the solid–liquid distribution coefficient (Kd), which relates the concentration of a substance in the liquid and solid phases under equilibrium conditions. The Kd value depends on both the physicochemical properties of the pollutant and the characteristics of the solid material. Therefore, this parameter must be determined for each matrix and treatment configuration, although some values have already been reported in the literature (Ternes et al. 2004; Carballa et al. 2008). In general, log Kd values below 2.0 (log Kd ≤ 2.0) indicate negligible sorption; values between 2.0 and 2.7 (2.0 ≤ log Kd ≤ 2.7) suggest a low sorption tendency; and values above 2.7 (log Kd ≥ 2.7) indicate a high probability of the compound associating with the solid phase (Ternes et al. 2004).

Furthermore, the sorption of contaminants onto biomass or suspended solids may result from both adsorption and absorption processes. Adsorption involves electrostatic interactions between positively charged functional groups of the pollutants and negatively charged sites on the sludge surface. Absorption, in turn, results from interactions between aliphatic and aromatic groups of pollutants in the liquid phase and the lipophilic cell membranes of microorganisms or the lipid fractions of suspended solids (Wang et al. 2020; Suárez et al. 2008). Thus, absorption is governed by the lipophilic/hydrophilic character of the pollutants, generally estimated by the octanol–water partition coefficient (Kow). Compounds with log Kow values below 2.5 (log Kow ≤ 2.5) are considered highly hydrophilic and have a low probability of being absorbed by biomass or suspended solids. Values between 2.5 and 4.0 (2.5 ≤ log Kow ≤ 4.0) indicate a moderate sorption tendency, whereas values above 4.0 (log Kow ≥ 4.0) characterize highly hydrophobic compounds with a strong propensity for absorption into solid matrices (Chakraborty et al. 2020; Rogers 1996).

However, since many pharmaceuticals contain polar functional groups (such as carboxyl, aldehyde, or amine groups) capable of interacting with specific moieties of organic matter, log Kow values alone are not always suitable for accurately predicting sorption behavior. In such cases, it is more appropriate to use the experimentally determined solid–liquid distribution coefficient (Kd) for each specific treatment system (Ternes et al. 2004).

Regarding biodegradation, which is considered the most important mechanism for micropollutant removal in MBR systems, contaminants can be degraded by enzymes produced by the microbial community via co-metabolism, or they can serve as a substrate, acting as a carbon source for microbial growth and maintenance (metabolism) (Tran et al. 2013). Studies indicate that biodegradation generally follows pseudo–first-order kinetics, with the biological transformation rate (Kbio) of the micropollutant being directly proportional to its concentration in the liquid phase and the biomass concentration in the system (assumed to be in excess and constant for calculation purposes).

Joss et al. (2006) conducted experiments to determine the Kbio values of several emerging contaminants. Aerobic sludge (0.5 g VSS L⁻^1^; VSS = volatile suspended solids) from an activated sludge system with solids retention times ranging from 10 to 15 days was used. Micropollutants were added at concentrations similar to those observed in sanitary sewage (3 μg L⁻^1^), and Kbio values were obtained by monitoring the decay of these compounds using a pseudo–first-order kinetic model. The results allowed the classification of pharmaceuticals and endocrine disruptors according to their biodegradability. Substances with Kbio < 0.1 L gSS⁻^1^ d⁻^1^ showed low removal via biodegradation (maximum efficiency < 20%), those with 0.1 ≤ Kbio ≤ 10 L gSS⁻^1^ d⁻^1^ could be partially biodegraded (removal between 20–90%), and compounds with Kbio > 10 L gSS⁻^1^ d⁻^1^ were expected to undergo high biological degradation (> 90%) (Joss et al. 2006).

In general, the removal of emerging contaminants in the investigated WRRF can be interpreted as the result of the combined action of the different treatment stages. The anaerobic and anoxic units may favor the transformation of some compounds under oxygen-limited conditions, while the aerobic stage is generally associated with the biodegradation of more readily biodegradable contaminants. The membrane bioreactor, in turn, improves effluent quality by retaining suspended solids and biomass and may also influence the removal of compounds associated with the solid phase. Thus, the removal patterns observed along the treatment system likely reflect the combined contribution of biological transformation, sorption, and membrane retention, depending on the characteristics of each contaminant.

Drug removal mechanisms are not necessarily related to therapeutic class. Therefore, the removal and behavior exhibited by the contaminants investigated in this study were classified into three groups: compounds showing high overall removal (> 97.0%), moderate removal (60.0–70.0%), and low removal or persistent behavior (< 21.0%). The classification of compounds into removal categories was adopted as a descriptive approach to facilitate the discussion of the observed patterns. These categories were defined based on the removal ranges obtained in the present study and do not represent statistically defined thresholds.

**Fig. 3:**
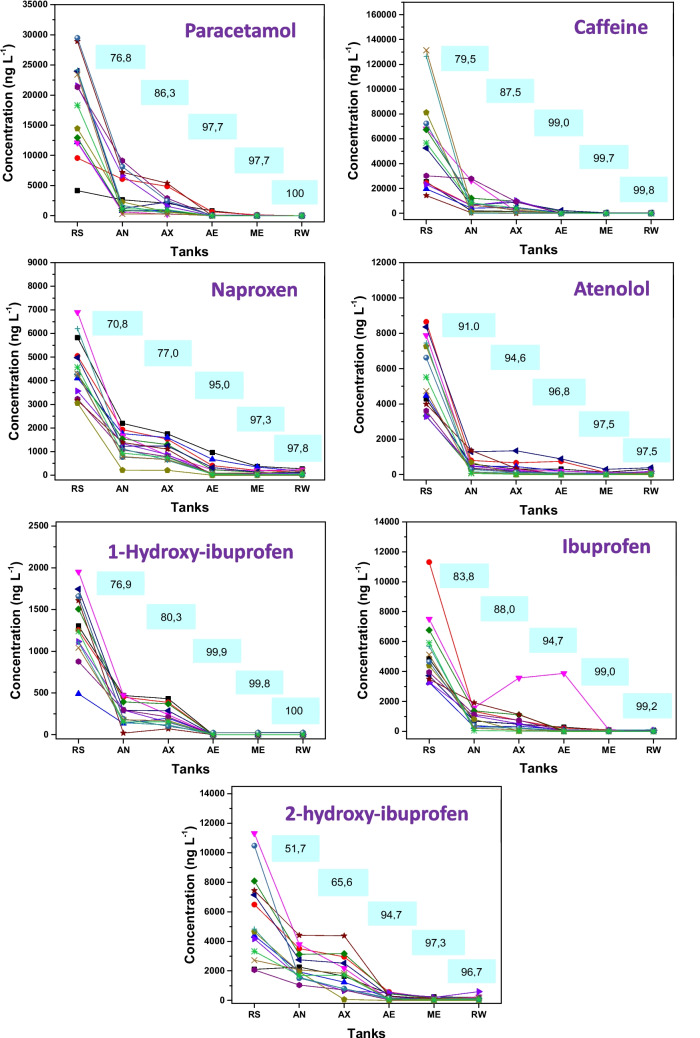
Removal behavior of PAR, CAF, NPX, ATL, IBU, IBU_1OH, and IBU_2OH during sewage treatment. RS: raw sewage; AN: anaerobic tank; AX: anoxic tank; AE: aeration tank; ME: membrane tank; RW: reclaimed water. The values shown in the text boxes represent the removal efficiencies (%) obtained at each treatment stage. Each line corresponds to one sampling campaign (*n* = 14)

The group of compounds with the highest average removal values (calculated based on raw sewage and treated effluent) includes paracetamol (100.0% ± 0.00%), caffeine (99.8% ± 0.28%), ibuprofen (99.2% ± 0.94%) and their metabolites 1-hydroxyibuprofen (100.0% ± 0.40%) and 2-hydroxyibuprofen (96.7% ± 4.14%), naproxen (97.8% ± 1.76%), and atenolol (97.5% ± 2.38%). The removal behavior and the average values obtained in each tank (calculated relative to raw sewage) are shown in Fig. 3.

In addition to the high removal efficiencies, these contaminants exhibited similar degradation patterns throughout the treatment process, with removals exceeding 50.0% already in the anaerobic tank. These compounds are expected to be predominantly present in the aqueous phase due to their molecular characteristics, physicochemical properties, and the operational pH of the treatment plant. Biological degradation is the main pathway for the removal of these micropollutants during sewage treatment (Verlicchi et al. 2012).

Paracetamol, for example, is a neutral compound with a low log Kow value (0.46), resulting in high molecular hydrophilicity; consequently, during treatment, it remains in the aqueous phase with a low tendency to sorb onto solid matrices. According to Tambosi et al. (2010), the high removal of this compound is related to its structure, which allows unrestricted access of bacteria and enzymes to the sterically unprotected molecule. Furthermore, the contaminant has a high Kbio value (106–240 L gSS⁻^1^ d⁻^1^), with biological removal above 90% expected (Joss et al. 2006).

Caffeine exhibits similar characteristics to paracetamol regarding molecular neutrality, low log Kow (−0.07), and high biodegradability. Gurung et al. (2019) reported an average removal efficiency above 99.7% for caffeine, a value close to that obtained in this study (99.8%) (Gurung et al. 2019). In the cited work, the removal of several emerging organic contaminants in municipal wastewater treated by pilot-scale MBR was investigated, testing two different solids retention times (60 and 21 days). The results demonstrated effective attenuation of caffeine even at a relatively short retention time. High removal values for paracetamol and caffeine are also reported in other studies (Phan et al. 2015; Snyder et al. 2007).

Ibuprofen and naproxen are acidic compounds (pKa 4.91 for ibuprofen and 4.15 for naproxen) and, therefore, it would be relevant to evaluate their sorption tendency in sludge using the solid–liquid distribution coefficient (Kd). In the work by Gurung et al. (2019), log Kd values were determined in the MBR system for several emerging contaminants, with values close to 4.0 L kg⁻^1^ for ibuprofen and naproxen (Gurung et al. 2019). The high log Kd values indicate a high probability of sorption of these micropollutants onto sludge. However, considering the wastewater pH 7.8 observed in the plant tanks, these acidic compounds are predominantly present in their ionized form. Based on the Henderson–Hasselbalch equation, more than 99.9% of ibuprofen and naproxen are expected to be deprotonated under these conditions. In this state, the negative charges of the pollutants repel the negatively charged sludge surface, preventing sorption and favoring their persistence in the aqueous phase. According to Quintana et al. (2005), co-metabolic biodegradation is the main mechanism for the removal of ibuprofen and naproxen in sewage treatment plants (Quintana et al. 2005).

Similar removal values for these compounds were obtained in the study by Kimura et al. (2005), in which drug removal was evaluated using two MBR systems: one conventional and one with pre-treatment (coagulation and sedimentation). The experiments were performed on a pilot scale using municipal wastewater. The removal of the investigated compounds was also compared with conventional activated sludge treatment (CAS) using the same municipal wastewater. The results showed that ibuprofen is highly susceptible to biological treatment, exhibiting high biodegradability with removal close to 100%, regardless of the system used (MBR or CAS). Regarding naproxen removal, the MBR with pre-treatment showed a slight increase in removal efficiency compared to the conventional MBR, with removal close to 100% in this system (Kimura et al. 2005).

High removal efficiency was also observed for atenolol at the end of sewage treatment, and similar results can be found in other studies (Jelic et al. 2011; Behera et al. 2011; Tadkaew et al. 2010). Stereoselective biological degradation is considered the main mechanism for removing this micropollutant in MBR systems (Nikolai et al. 2006; Maurer et al. 2007). In addition, these compounds, which showed high removal efficiencies, were not detected in the sludge, suggesting that sorption was not a major removal pathway under the investigated conditions.

**Fig. 4:**
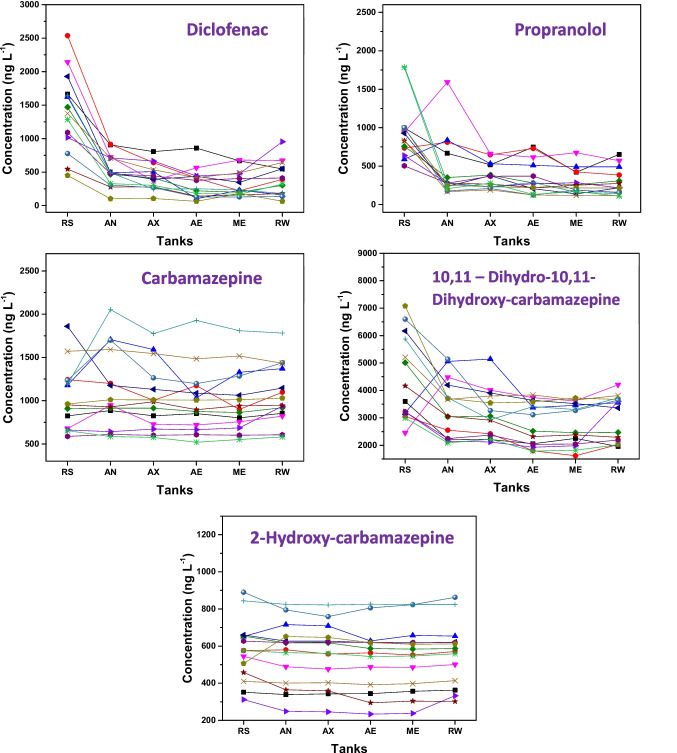
Removal behavior of DCL, PRP, CBZ, CBZ_DIOH, and CBZ_2OH during sewage treatment. RS: raw sewage; AN: anaerobic tank; AX: anoxic tank; AE: aeration tank; ME: membrane tank; RW: reclaimed water. The values shown in the text boxes represent the removal efficiencies (%) obtained at each treatment stage. Each line corresponds to one sampling campaign (*n* = 14)

The group of compounds exhibiting moderate removal includes diclofenac and propranolol (Fig. 4). Diclofenac is an acidic compound (pKa: 4.14) and, considering the wastewater pH 7.8, is likely present in its ionized form. Thus, this pollutant has a low tendency for adsorption onto sludge and a limited biodegradation potential, reflected by its low Kbio (0.1 L gSS⁻^1^ d⁻^1^, calculated for an MBR system). However, a removal of 70.5% ± 12.45% was observed at the end of the sewage treatment for diclofenac.

Removal efficiencies reported in the literature for this micropollutant vary widely, ranging from negligible removal to values exceeding 70.0% (Zwiener and Frimmel 2003). Zwiener and Frimmel (2003) conducted biodegradation tests of diclofenac in a pilot-scale sewage treatment plant with three consecutive stages (denitrification, activated sludge, and sedimentation) and also evaluated reactors with aerobic and anoxic biofilms. The initial diclofenac concentration used in the tests was 10 µg L⁻^1^, and during 55 h of treatment at the pilot plant, no degradation of the drug was observed. Similar results were found in the reactor with aerobic biofilm. However, diclofenac was better removed in the reactor with anoxic biofilm, with removal efficiencies ranging from 34.0 to 38.0%. It was thus concluded that the anoxic–oxic ratio may influence diclofenac removal, which may partly explain the wide range of removal observed for this compound. This finding is consistent with the removal behavior obtained for diclofenac in the present study, where approximately 60.0% removal was observed in the anoxic tanks. In the subsequent tanks, under aerobic conditions, no significant additional decrease in the concentration of this micropollutant was observed (as shown in Fig. 4).

Regarding propranolol, its molecule has a basic character (pKa: 9.42) and is likely protonated in solution (given the wastewater pH 7.8); therefore, it tends to adsorb onto sludge. In this study, propranolol was detected in sludge at 95 ng g⁻^1^. In addition, previous studies suggest that biodegradation is also a removal mechanism for this compound, which showed an average final removal of 61.5% ± 16.12% in the present study (Joss et al. 2006).

Carbamazepine and its metabolites exhibited low removal during sewage treatment. At the end of the process, carbamazepine showed a negative removal rate (−7.36% ± 23.13%), indicating that its concentration in the treated effluent exceeded that measured in raw sewage (Fig. 4). This behavior is commonly attributed to back-transformation processes, in which carbamazepine is excreted predominantly as conjugated metabolites that undergo enzymatic cleavage during biological treatment, regenerating the parent compound. The deconjugation of glucuronides and other transformation products explains the apparent increase in carbamazepine concentration along the treatment train and reinforces its well-documented persistence in wastewater treatment systems, supporting its use as a chemical marker of treatment efficiency (Gurung et al. 2019).

From a physicochemical perspective, carbamazepine is a neutral compound with a log Kow of approximately 2.47, indicating moderate hydrophobicity and a limited tendency to adsorb onto sludge. In addition, it exhibits a low biological transformation rate (Kbio ≈ 0.1 L gSS⁻^1^ d⁻^1^, calculated for MBR systems), which results in a low biodegradation potential. Furthermore, the presence of an electron-withdrawing amide group in its molecular structure hinders enzymatic degradation (Hai et al. 2011). Together, these properties explain the persistence of carbamazepine during sewage treatment and further justify its use as a reliable chemical marker of treatment efficiency.

To further support this interpretation, the robustness of the observed negative removal of carbamazepine was evaluated considering the variability across monitoring campaigns and analytical reliability. The results are based on multiple monitoring campaigns (*n* = 14), and the variability observed across sampling events indicates that this behavior is consistent rather than driven by isolated measurements. All samples were analyzed in triplicate, and method performance parameters are provided in the Supplementary Material, supporting the reliability of the analytical data. In addition, carbamazepine and its metabolites were not detected in the sludge, suggesting that sorption did not play a major role in their removal under the investigated conditions.

**Fig. 5:**
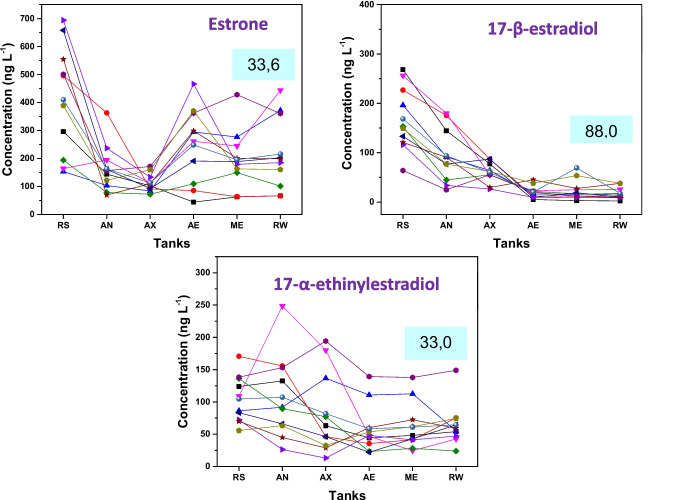
Removal behavior of E1, E2, and EE2 during sewage treatment. RS: raw sewage; AN: anaerobic tank; AX: anoxic tank; AE: aeration tank; ME: membrane tank; RW: reclaimed water. The values shown in the text boxes represent the removal efficiencies (%) obtained at each treatment stage. Each line corresponds to one sampling campaign (*n* = 11)

Regarding estrogenic compounds, removals of 88.0% ± 9.57% for 17β-estradiol, 33.6% ± 18.16% for estrone, and 33.0% ± 24.99% for 17α-ethinylestradiol and the removal profiles of these compounds can be seen in Fig. 5. Removal values of estrogens in the literature vary considerably, regardless of the type of treatment applied (anaerobic, aerobic, or others). Variations in sewage treatment processes and operating conditions are considered the main reasons for fluctuations in estrogen removal efficiencies. Typically, low removal is associated with high organic loading rates and short hydraulic and solids retention times. Plants operating with solids retention times longer than 10 days and processes with effective nitrification tend to achieve better estrogen removal rates (Liyanage et al. 2024; Racz and Goel 2010).

Although some removal occurs via sorption, biodegradation is the main mechanism for estrogen removal in sewage (Liyanage et al. 2024). Biodegradation can occur via metabolism, that is, as a carbon source for heterotrophic bacteria, as co-metabolism with nitrifying biomass, or through other types of co-metabolism. In general, natural estrogens, especially 17β-estradiol, are readily biodegradable (Ternes et al. 1999). However, 17α-ethinylestradiol is not easily removed by biological processes, as the ethynyl group in the molecular structure constitutes a steric hindrance to enzymatic action (Racz and Goel 2010). These facts were consistent with the results obtained in this study, in which 17β-estradiol showed the highest removal (88% ± 9.57%) among the estrogens studied, while 17α-ethinylestradiol exhibited low removal (33% ± 24.99%).

Regarding the behavior of these compounds during sewage treatment, an increase in estrone concentration was observed in the oxic tanks, which may be related to the deconjugation process. Estrone can be excreted in its conjugated form, and the bacteria present in the medium, particularly *Escherichia coli*, are capable of deconjugating it, restoring its free form. Another factor that may explain the increase in estrone concentration is the partial oxidation of other estrogenic hormones (mainly 17β-estradiol and estriol) into estrone. It is observed that the concentration of 17β-estradiol decreased in the oxic tanks while estrone concentration increased. Thus, with the increase in estrone concentration during sewage treatment, this compound exhibited a low removal rate (33.6% ± 18.16%) at the end of the process (Kumar et al. 2012; Alvarino et al. 2014).

### Occurrence of emerging contaminants in surface waters and comparison with reclaimed water produced by the WRRF

The analysis of contaminants was carried out in surface waters used for urban water supply, sampled from the Ribeirão Piraí, Atibaia, Jundiaí, and Capivari rivers, all located in São Paulo State, Brazil. The results showed that the Ribeirão Piraí River presented the lowest concentrations of the investigated compounds (Table 1). In this river, caffeine was detected at the highest concentration (1280 ng L⁻^1^). This comparatively high value, relative to other pharmaceuticals, may be associated with the widespread consumption of caffeine in beverages and pharmaceutical products. Additionally, concentrations of 7 ng L⁻^1^ for naproxen, 14 ng L⁻^1^ for ibuprofen, 13 ng L⁻^1^ for atenolol, 21 ng L⁻^1^ for propranolol, 84 ng L⁻^1^ for carbamazepine, and 36 ng L⁻^1^ for diclofenac were measured. Estrogens were below the detection limit, and carbamazepine metabolites, 2-hydroxycarbamazepine (30 ng L⁻^1^), and 10,11-dihydro-10,11-dihydroxycarbamazepine (91 ng L⁻^1^), as well as ibuprofen metabolites 1-hydroxyibuprofen (30 ng L⁻^1^) and 2-hydroxyibuprofen (86 ng L⁻^1^), were also found in Ribeirão Piraí.

**Table 1:** Concentrations of contaminants in rivers and reclaimed water produced by the WRRF

Compound	Piraí (ng L^−1^)	Atibaia (ng L^−1^)	Capivari (ng L^−1^)	Jundiaí (ng L^−1^)	Reclaimed water (ng L^−1^)
CAF	1280	3023	24,015	38,994	86
DCL	36	22	52	102	358
IBU	14	82	649	367	48
IBU_1OH	30	73	169	131	< LOD
IBU_2OH	86	113	247	303	152
NPX	7	46	328	155	107
PAR	41	252	164	354	< LOD
ATL	13	120	307	173	129
PRP	21	8	253	45	287
CBZ	84	108	613	367	1069
CBZ_2OH	30	15	132	233	640
CBZ_DIOH	91	119	940	735	3040
E1	< LOD	3	< LOQ	2	162
E2	< LOD	5	< LOQ	5	16
EE2	< LOD	< LOQ	< LOQ	9	54

LOD and LOQ values for all analytes are provided in the Supplementary Material (Table S2). Concentrations reported as  < LOD were treated as zero for statistical calculations

The Capivari and Jundiaí rivers exhibited the highest concentrations of the investigated compounds, likely due to their location in densely populated and industrialized regions, which receive domestic and industrial wastewater discharges. Caffeine was the most abundant compound, with concentrations of 24,015 ng L⁻^1^ in the Capivari River and 38,994 ng L⁻^1^ in the Jundiaí River.

Among anti-inflammatory drugs, ibuprofen (649 ng L⁻^1^), its metabolite 1-hydroxyibuprofen (169 ng L⁻^1^), and naproxen (328 ng L⁻^1^) showed higher concentrations in the Capivari River than in the Jundiaí River (367 ng L⁻^1^, 131 ng L⁻^1^, and 155 ng L⁻^1^, respectively). Conversely, diclofenac (102 ng L⁻^1^), 2-hydroxyibuprofen (303 ng L⁻^1^), and paracetamol (354 ng L⁻^1^) were detected at higher concentrations in the Jundiaí River, while their concentrations in the Capivari River were 52 ng L⁻^1^, 247 ng L⁻^1^, and 164 ng L⁻^1^, respectively.

The β-blockers atenolol and propranolol were present at higher concentrations in the Capivari River (307 ng L⁻^1^ and 253 ng L⁻^1^, respectively) compared to the Jundiaí River (173 ng L⁻^1^ and 45 ng L⁻^1^, respectively). In the Capivari River, carbamazepine and its metabolites, 2-hydroxycarbamazepine and CBZ_DIOH, were detected at concentrations of 613 ng L⁻^1^, 132 ng L⁻^1^, and 940 ng L⁻^1^, respectively. In the Jundiaí River, concentrations of 367 ng L⁻^1^, 233 ng L⁻^1^, and 735 ng L⁻^1^ were observed for the same compounds. Estrogens were not quantified in the Capivari River, whereas estrone (2 ng L⁻^1^), 17β-estradiol (5 ng L⁻^1^), and 17α-ethinylestradiol (9 ng L⁻^1^) were detected in the Jundiaí River.

In the Atibaia River, caffeine was again the compound detected at the highest concentration (3023 ng L⁻^1^). Among anti-inflammatory drugs, paracetamol presented the highest concentration (252 ng L⁻^1^), followed by ibuprofen (82 ng L⁻^1^), its metabolites 2-hydroxyibuprofen (113 ng L⁻^1^) and 1-hydroxyibuprofen (73 ng L⁻^1^), and naproxen (46 ng L⁻^1^). Diclofenac was detected at the lowest concentration (22 ng L⁻^1^). Atenolol (120 ng L⁻^1^) was approximately 15 times more concentrated than propranolol (8 ng L⁻^1^) in this river. 17α-ethinylestradiol was not detected, while estrone and 17β-estradiol were quantified at 3 ng L⁻^1^ and 5 ng L⁻^1^, respectively. Carbamazepine (108 ng L⁻^1^) and its metabolite CBZ_DIOH (119 ng L⁻^1^) were detected at higher concentrations than 2-hydroxycarbamazepine (15 ng L⁻^1^).

When comparing the concentrations of contaminants in surface waters and in the reclaimed water produced by the WRRF (Table 1), it is evident that caffeine removal during treatment was highly effective in the WRRF, resulting in a low concentration in the reclaimed water (85 ng L⁻^1^), significantly lower than those observed in surface waters. Furthermore, 1-hydroxyibuprofen and paracetamol were not detected in the reclaimed water.

In the reclaimed water, naproxen (107 ng L⁻^1^), atenolol (129 ng L⁻^1^), ibuprofen (48 ng L⁻^1^), and its metabolite 2-hydroxyibuprofen (152 ng L⁻^1^) were detected at lower concentrations than those measured in the Capivari and Jundiaí rivers.

However, some recalcitrant compounds remaining after biological treatment were detected in the reclaimed water at higher concentrations than those observed in the rivers used as water sources for different cities. Among these compounds, diclofenac (358 ng L⁻^1^), propranolol (287 ng L⁻^1^), carbamazepine (1069 ng L⁻^1^), and its metabolites 2-hydroxycarbamazepine (640 ng L⁻^1^) and CBZ_DIOH (3040 ng L⁻^1^) were quantified.

Based on these results, complementary treatment processes are required when stricter water quality criteria for emerging contaminants are needed for the intended reuse application. Nevertheless, the effluent produced by the membrane bioreactor (MBR) system showed high treatment performance with respect to conventional wastewater quality parameters, including the removal of total organic load, caffeine, nutrients, total suspended solids (TSS), biochemical oxygen demand (BOD), total Kjeldahl nitrogen (TKN), and turbidity (NTU), as shown in Table 2. Therefore, the reclaimed water can be considered of good quality with respect to these conventional parameters, although the persistence of some recalcitrant contaminants indicates that additional polishing steps may be necessary depending on the intended reuse and associated exposure scenario.

**Table 2:** Summary of physicochemical parameters obtained from operational monitoring data provided by the wastewater treatment plant during the same sampling period of the emerging contaminants

Parameter	Raw sewage		Reclaimed water		Average removal (%)
	Range	Average	Range	Average	
BOD (mg L^−1^)	185.0–572.0	373.0	0.1–1.4	< 1	> 99.7%
TKN (mg-N L^−1^)	15.4–123.0	70.5	0.01–2.7	0.9	98.7%
Nitrate (mg-N L^−1^)	–	–	0.02–13.5	7.8	–
Phosphate (mg-P L^−1^)	4.8–17.0	8.0	0.10–6.8	2.0	71.0%
TSS (mg L^−1^)	196.0–720.0	314	0.6–4	< 2.5	> 99.5%
Turbidity (NTU)	–	–	0.1–0.5	0.2	–

*BOD* biochemical oxygen demand, *TKN* total Kjeldahl nitrogen, *TSS* total suspended solids

In a broader context, the comparison with surface water samples was included to provide a regional reference for the occurrence of the investigated contaminants. However, this comparison should be interpreted with caution, since river samples are influenced by distinct hydrological conditions, dilution regimes, and environmental exposure scenarios that were not normalized in the present study. Therefore, these comparisons are presented as a qualitative and contextual reference, rather than as direct proof of treatment adequacy or environmental safety.

## Conclusions

Based on the results obtained, the presence of all contaminants investigated was confirmed in the raw sewage samples collected during the sampling campaigns. Regarding the removal of pharmaceuticals in the WRRF, paracetamol, caffeine, naproxen, atenolol, ibuprofen, and their metabolites (1-hydroxyibuprofen and 2-hydroxyibuprofen) were highly susceptible to biodegradation, achieving average removal efficiencies exceeding 90%. In addition, these compounds exhibited similar degradation profiles, with removal rates exceeding 50% already observed in the anaerobic tank.

Diclofenac and propranolol showed average removal efficiencies of 70.6% and 61.5%, respectively. Estrogenic compounds exhibited removals of 88.0% for 17β-estradiol, 33.6% for estrone, and 33.0% for 17α-ethinylestradiol.

Carbamazepine and its metabolites, 2-hydroxycarbamazepine and 10,11-dihydro-10,11-dihydroxycarbamazepine, reached the end of the treatment process with removal efficiencies of −7.36%, 5.7%, and 21.0%, respectively. The persistent behavior of carbamazepine reinforces its suitability as a chemical marker of sewage treatment efficiency.

Overall, the results demonstrate that the WRRF is effective in removing a wide range of biodegradable pharmaceuticals. In addition, the effluent produced by the membrane bioreactor (MBR) system showed good performance with respect to conventional wastewater quality parameters. However, the persistence of some recalcitrant compounds indicates that the suitability of the reclaimed water should be evaluated according to the intended reuse purpose and the associated exposure routes. In this context, advanced polishing technologies, such as ozonation, which promotes the oxidation of recalcitrant compounds; activated carbon adsorption, which enables the removal of micropollutants through surface interactions; and advanced oxidation processes (AOPs), which generate highly reactive species capable of degrading resistant contaminants, represent promising alternatives to improve the removal of persistent contaminants and to support safer and more sustainable wastewater reuse practices.

## Supplementary Information

Below is the link to the electronic supplementary material.

ESM1 (DOCX 28.6 KB)

## Data Availability

The datasets generated and analyzed during the current study, including raw concentration data and sampling campaign information (e.g., dates), are available from the corresponding author upon reasonable request due to their large volume. Quality assurance and quality control (QA/QC) data are provided in the Supplementary Material. The procedures used for removal calculations are described in the “Data treatment and removal calculation” section.
