# Supplementary material for: Removal of emerging contaminants in a full-scale bioreactor coupled with membrane filtration for reclaimed water production in São Paulo State, Brazil
Source: Environ Sci Pollut Res Int. 2026 Apr 29;33(15):7317–32. doi: 10.1007/s11356-026-37765-1 (PMC13156141; doi:10.1007/s11356-026-37765-1)
Supplement: Supplementary file 1 — (DOCX 28.6 KB) [file 11356_2026_37765_MOESM1_ESM.docx]

**SUPPLEMENTARY MATERIAL**

**Removal of Emerging Contaminants in a Full-Scale Bioreactor Coupled with Membrane Filtration for Reclaimed Water Production in São Paulo State, Brazil**

Rafaela Gonçalves Machado^1^*, Josilei da Silva Ferreira^1^, Guilherme Martins Grosseli^1^, Roberta Cerasi Urban^1^, Pedro Sergio Fadini^1^

¹Environmental Biogeochemistry Laboratory and Center for Environmental Diagnostics and Interventions/Department of Chemistry, Federal University of São Carlos, São Carlos, Brazil

______________________________________________________________________

*Corresponding author:

Rafaela Gonçalves Machado

E-mail: rafaela_machado08@hotmail.com

Telephone: 16 3509-1515

Fax: not available

**Quality Assurance and Quality Control (QA/QC)**

Quality assurance (QA) and quality control (QC) procedures included recovery assays, replicate analyses, calibration curve evaluation, and determination of method detection and quantification limits. Recovery experiments were performed in triplicate using treated effluent samples and sludge samples collected from the aeration tank. For aqueous samples, absolute recovery (R%) was calculated as the ratio between the peak area obtained from samples spiked with native analytes and their respective deuterated internal standards before solid-phase extraction (SPE) and the peak area obtained from samples fortified at the vial reconstitution step, according to Equation (1):

$R\left( \% \right)= \left( \frac{A_{pre-extration}}{A_{post-extration}} \right) x 100$ (Equation 1)

Where $"A_{\text{pre-extration}}"$is the peak area of the analyte in the sample fortified before extraction and $"A_{\text{post-extration}}"$is the peak area of the analyte in the sample fortified after extraction, at the reconstitution step. Relative recovery (RR%) was determined from the ratio between the peak areas of each analyte and its corresponding deuterated internal standard, as shown in Equation (2):

$\mathrm{RR}\left( \% \right)= \left( \frac{A_{native compound}}{A_{deuterated compound}} \right) x 100$ (Equation 2)

where $"A_{\text{native compound}}"$is the peak area of the native analyte and ${"A}_{\text{deuterated compound}}"$ is the peak area of the corresponding deuterated internal standard.

For sludge samples, absolute recovery was determined by comparing the peak area of samples fortified before ultrasound-assisted extraction with that of samples subjected to extraction and fortified only at the reconstitution step. Relative recovery was likewise assessed based on the analyte-to-internal-standard peak area ratio.

Calibration curves for LC–MS/MS and GC–MS analyses were prepared using internal standardization, with analyte concentrations ranging from 0.05 to 1200 µg L⁻¹ (17 calibration points), while internal standards were maintained at a fixed concentration of 50 µg L⁻¹. Linearity was evaluated using the coefficient of determination (R²), and values higher than 0.99 were considered acceptable. Limits of detection (LOD) and limits of quantification (LOQ) were calculated for each analyte from the calibration curve parameters according to Equations (3) and (4):

$LOD=3.3 x \frac{s}{a}$ (Equation 3)

$LOQ=10 x \frac{s}{a}$ (Equation 3)

where “s” is the estimated standard deviation of the response and “a” is the slope of the calibration curve. Final analyte concentrations were corrected according to the recovery values obtained during method validation.

For LC–MS/MS identification, quantification was based on two selected reaction monitoring (SRM) transitions per compound, according to European Commission Decision 2002/657/EC. The most intense transition was used for quantification and the second for confirmation. Identification criteria included agreement of retention time and consistency of ion ratios relative to calibration standards.

Overall, the validation results indicated satisfactory analytical performance for the determination of the investigated compounds in treated effluent and sludge matrices, with acceptable recoveries, good linearity, and adequate method sensitivity.

**Table S1.** Absolute recovery (R%), relative recovery (RR%), and relative standard deviation (RSD, in parentheses) for treated effluent and sludge samples.

| **Analyte** | **Treated effluent R%** | **Treated effluent RR%** | **Sludge R%** | **Sludge RR%** |
| --- | --- | --- | --- | --- |
| Caffeine | 106 (9) | 99 (9) | 92 (4) | 103 (7) |
| Diclofenac | 100 (9) | 105 (7) | 61 (5) | 77 (8) |
| Ibuprofen | 98 (8) | 108 (7) | 71 (7) | 83 (6) |
| 1-Hydroxyibuprofen | 108 (6) | 96 (5) | 102 (5) | 92 (3) |
| 2-Hydroxyibuprofen | 92 (8) | 104 (8) | 78 (5) | 102 (5) |
| Naproxen | 101 (4) | 99 (6) | 69 (7) | 84 (9) |
| Paracetamol | 107 (7) | 105 (8) | 80 (9) | 79 (7) |
| Atenolol | 101 (5) | 106 (8) | 85 (4) | 95 (9) |
| Propranolol | 99 (3) | 96 (4) | 64 (9) | 69 (3) |
| Carbamazepine | 111 (6) | 105 (6) | 106 (7) | 99 (4) |
| 2-Hydroxycarbamazepine | 101 (5) | 89 (6) | 84 (4) | 101 (9) |
| 10,11-Dihydro-10,11-dihydroxycarbamazepine | 107 (3) | 96 (5) | 79 (10) | 97 (4) |
| Estrone | 93 (5) | 88 (6) | * | * |
| 17β-Estradiol | 99 (8) | 83 (4) | * | * |
| 17α-Ethinylestradiol | 87 (7) | 92 (5) | * | * |

* Recovery for estrogens in sludge could not be determined.

**Table S2.** Calibration performance and analytical sensitivity parameters for the LC–MS/MS and GC–MS methods, including calibration range, number of calibration points, internal standard concentration, correlation coefficient (R²), limits of quantification (LOQ), and limits of detection (LOD).

| **Parameter** | **Value** |
| --- | --- |
| Calibration range | 0.05–1200 µg L⁻¹ |
| Number of calibration points | 17 |
| Internal standard concentration | 50 µg L⁻¹ |
| Coefficient of determination (R²) range | 0.991–0.997 |
| LC–MS/MS LOQ range | 3.32–7.92 ng L⁻¹ |
| LC–MS/MS LOD range | 1.07–2.63 ng L⁻¹ |
| GC–MS LOQ range | 1.40–4.65 ng L⁻¹ |
| GC–MS LOD range | 0.39–1.61 ng L⁻¹ |

**Table S3.** Selected reaction monitoring (SRM) transitions and instrumental parameters used for analyte determination by LC–MS/MS, including cone voltage and collision energy.

| **Compound** | **SRM 1** | **Cone voltage (V)** | **Collision energy (eV)** | **SRM 2** | **Collision energy (eV)** |
| --- | --- | --- | --- | --- | --- |
| *Analyzed in positive ionization mode (+)* |  |  |  |  |  |
| Caffeine | 195.0 → 138.1 | 35 | 20 | 195.0 → 42.0 | 30 |
| Caffeine-d₃ | 198.0 → 138.0 | 35 | 22 | - | - |
| Atenolol | 267.0 → 145.0 | 32 | 30 | 167.0 → 190.0 | 20 |
| Atenolol-d₇ | 274.0 → 145.0 | 32 | 28 | - | - |
| Carbamazepine | 237.1 → 194.1 | 33 | 20 | 237.1 → 192.0 | 20 |
| Carbamazepine-d₁₀ | 247.0 → 204.0 | 33 | 25 | - | - |
| Propranolol | 260.1 → 72.0 | 34 | 24 | 260.1 → 116.0 | 22 |
| Propranolol-d₇ | 267.2 → 72.0 | 34 | 25 | - | - |
| 2-Hydroxycarbamazepine | 253.2 → 208.2 | 20 | 20 | 253.2 → 210.2 | 20 |
| CBZ-DiOH | 271.0 → 236.0 | 15 | 9 | 271.0 → 253.0 | 9 |
|  |  |  |  |  |  |
| *Analyzed in negative ionization mode (−)* |  |  |  |  |  |
| Naproxen | 229.0 → 170.1 | 15 | 15 | 229.0 → 185.1 | 8 |
| Naproxen-d₃ | 232.0 → 188.0 | 12 | 8 | - | - |
| Ibuprofen | 205.1 → 161.1 | 15 | 8 | 205.1 | - |
| Ibuprofen-d₃ | 208.0 → 164.0 | 15 | 8 | - | - |
| 1-Hydroxyibuprofen | 221.3 → 159.3 | 15 | 8 | 221.3 → 177.3 | 10 |
| 2-Hydroxyibuprofen | 221.3 → 177.3 | 15 | 8 |  |  |
| Diclofenac | 294.0 → 250.1 | 20 | 12 | 296.0 → 252.0 | 12 |
| Diclofenac-d₄ | 298.0 → 254.0 | 20 | 12 | - | - |
